# Real-world experience of patients with multiple myeloma receiving ide-cel after a prior BCMA-targeted therapy

**DOI:** 10.1038/s41408-023-00886-8

**Published:** 2023-08-09

**Authors:** Christopher J. Ferreri, Michelle A. T. Hildebrandt, Hamza Hashmi, Leyla O. Shune, Joseph P. McGuirk, Douglas W. Sborov, Charlotte B. Wagner, M. Hakan Kocoglu, Aaron Rapoport, Shebli Atrash, Peter M. Voorhees, Jack Khouri, Danai Dima, Aimaz Afrough, Gurbakhash Kaur, Larry D. Anderson, Gary Simmons, James A. Davis, Nilesh Kalariya, Lauren C. Peres, Yi Lin, Murali Janakiram, Omar Nadeem, Melissa Alsina, Frederick L. Locke, Surbhi Sidana, Doris K. Hansen, Krina K. Patel, Omar Alexis Castaneda Puglianini

**Affiliations:** 1 The University of Texas MD Anderson Cancer Center, Houston, TX USA; 2 Medical University of South Carolina, Charleston, SC USA; 3 The University of Kansas Medical Center, Kansas City, KS USA; 4 The University of Utah Huntsman Cancer Institute, Salt Lake City, UT USA; 5 University of Maryland Marlene and Stewart Greenebaum Comprehensive Cancer Center, Baltimore, MD USA; 6 Levine Cancer Institute, Charlotte, NC USA; 7 Cleveland Clinic Taussig Cancer Center, Cleveland, OH USA; 8 Myeloma, Waldenstrom’s, and Amyloidosis Program, Simmons Comprehensive Cancer Center, UT Southwestern Medical Center, Dallas, TX USA; 9 Virginia Commonwealth University Massey Cancer Center, Richmond, VA USA; 10 H. Lee Moffitt Cancer Center and Research Institute, Tampa, FL USA; 11 Mayo Clinic, Rochester, MN USA; 12 City of Hope, Duarte, CA USA; 13 Dana-Farber Cancer Institute, Boston, MA USA; 14 Stanford University School of Medicine, Stanford, CA USA

**Keywords:** Myeloma, Myeloma, Cancer immunotherapy, Cancer therapeutic resistance

## Abstract

Most patients with multiple myeloma experience disease relapse after treatment with a B-cell maturation antigen-targeted therapy (BCMA-TT), and data describing outcomes for patients treated with sequential BCMA-TT are limited. We analyzed clinical outcomes for patients infused with standard-of-care idecabtagene vicleucel, an anti-BCMA chimeric antigen receptor (CAR) T-cell therapy, at 11 US medical centers. A total of 50 patients with prior BCMA-TT exposure (38 antibody-drug conjugate, 7 bispecific, 5 CAR T) and 153 patients with no prior BCMA-TT were infused with ide-cel, with a median follow-up duration of 4.5 and 6.0 months, respectively. Safety outcomes between cohorts were comparable. The prior BCMA-TT cohort had a lower overall response rate (74% versus 88%; *p* = 0.021), median duration of response (7.4 versus 9.6 months; *p* = 0.03), and median progression-free survival (3.2 months versus 9.0 months; *p* = 0.0002) compared to the cohort without prior BCMA-TT. All five patients who received a prior anti-BCMA CAR T responded to ide-cel, and survival outcomes were best for this subgroup. In conclusion, treatment with ide-cel yielded meaningful clinical responses in real-world patients exposed to a prior BCMA-TT, though response rates and durability were suboptimal compared to those not treated with a prior BCMA-TT.

## Introduction

Prior to the development of therapies targeting the B-cell maturation antigen (BCMA), patients with multiple myeloma refractory to immunomodulatory agents (IMiD), proteasome inhibitors (PI), and anti-CD38 monoclonal antibodies had limited therapeutic options and an estimated median overall survival (OS) of 5.6–13 months [1, 2]. The antibody-drug conjugate (ADC) belantamab mafodotin was the first BCMA-targeted therapy to receive accelerated regulatory approval in August 2020, but has since been withdrawn from commercial use [3, 4]. The BCMA-targeted chimeric antigen receptor (CAR) T-cell therapies idecabtagene vicleucel (ide-cel) and ciltacabtagene autoleucel (cilta-cel) have demonstrated unprecedented efficacy, and each received regulatory approval for use in patients with ≥4 prior lines of therapy including an IMiD, PI, and anti-CD38 monoclonal antibody in March 2021 and February 2022 respectively [5, 6]. Bispecific T-cell redirecting antibody therapies have also demonstrated impressive efficacy in relapsed/refractory multiple myeloma (RRMM). The first agent of this class to receive regulatory approval was teclistamab (October 2022), and other trials of BCMA-targeted bispecifics have shown promising results [7, 8].

Given the emergence of BCMA-targeted therapies with three distinct mechanisms of action, treatment of patients with disease progression after receiving a BCMA-targeted therapy (BCMA-TT) has become an unmet need. The registrational trials for the approved BCMA-TT excluded patients who had received a prior BCMA-TT, resulting in limited data characterizing outcomes for such patients [3, 5–7]. Recent clinical trials involving the bispecific antibodies teclistamab and elranatamab, as well as the CAR T cilta-cel, have included small cohorts of patients who received a prior BCMA-TT and the overall response rate (ORR) was above 50% with all three agents [9–11].

In the pivotal phase 2 KarMMa trial, patients infused with ide-cel achieved an ORR of 73%, ≥ complete response (CR) rate of 33%, median progression-free survival (PFS) of 8.6 months, and median OS of 24.8 months [5, 12]. This consortium has previously published retrospective outcomes for the largest cohort of patients treated with commercially available ide-cel in a real-world setting. While efficacy outcomes were generally comparable with results from the KarMMa study, treatment with a prior BCMA-TT was an independent predictor of inferior PFS [13]. The goal of this study was to perform an in-depth evaluation of the outcomes for patients who had received a prior BCMA-TT before commercial ide-cel.

## Methods

### Study treatment and data collection

This was a retrospective multicenter observational study of patients with RRMM for whom treatment with commercial ide-cel was planned at one of 11 US medical centers. Each center obtained independent institutional review board approval and informed consent per institutional requirements. While data was collected for all patients who underwent leukapheresis with the intent to manufacture ide-cel from April 1, 2021 to May 1, 2022, patients included in this study were those who underwent ide-cel infusion and had at least a day 30 response assessment completed by the time of data cut-off. Patients who died after ide-cel infusion but prior to response assessment were included in the safety and survival analyses, but not in the response analysis.

Following leukapheresis, there were no restrictions on the use of bridging or radiation therapy. Lymphodepleting chemotherapy was administered on days -5 through -3 with cyclophosphamide 300 mg/m^2^ and fludarabine renally-dose adjusted per institutional guidelines. Cytokine release syndrome (CRS) and immune-effector cell-associated neurotoxicity syndrome (ICANS) were graded as per the American Society for Transplantation and Cellular Therapy (ASTCT) criteria [14]. Hematologic toxicities were graded using the National Cancer Institute Common Terminology Criteria for Adverse Events (NCI-CTCAE), Version 5.0. Infectious disease prophylaxis, use of growth factors, and treatment of CRS and ICANS were managed per institutional guidelines. Each institution graded response to therapy as per International Myeloma Working Group (IMWG) criteria; however, confirmatory testing and imaging to confirm CR for patients with extramedullary disease were not mandated due to the retrospective study design [15].

### Statistical analysis

Baseline characteristics and outcomes related to efficacy and safety were summarized with descriptive statistics. Differences between groups were evaluated using chi-squared or Fisher’s exact tests for categorical variables, or Kruskal-Wallis rank-sum tests for continuous variables. Definitions for duration of response (DOR), PFS, and OS are provided in Appendix 1, as are the methods for the multivariable logistic regression analysis examining the association of prior BCMA-TT with best ORR (≥partial response [PR] versus < PR), and best CR rate (≥CR versus < CR). The Kaplan–Meier method was used to estimate DOR, PFS, and OS, and survival outcomes amongst subgroups were compared using the log-rank test. Multivariable Cox proportional hazard regression models were used to analyze the association of prior BCMA-TT with PFS and OS while adjusting for the selected characteristics outlined in Appendix 1. Proportional hazards assumptions via Schoenfeld residuals were examined under these parameters with no deviations detected for the global tests. High-risk cytogenetics violated the proportional hazards assumption for the OS model and was included as a strata term in that model. All statistical tests were two-sided and *P*-values of <0.05 were considered significant. Statistical analyses were conducted using STATA 17 (StataCorp, College Station, TX).

## Results

### Patients and treatment

As of May 1, 2022, 239 patients underwent leukapheresis with the intention to manufacture ide-cel. Of the 56 patients who had received a prior BCMA-TT, five patients did not proceed with ide-cel infusion (one manufacturing failure and four due to interim progression/death), and one patient was pending infusion at data cutoff. Thus, 91% of patients who had received a prior BCMA-TT (50 of 55 evaluable) were able to complete ide-cel infusion (Supplemental Figs. 1 and 2).

Patient baseline characteristics are presented in Table 1 stratified by the cohort who had received a prior BCMA-TT (*n* = 50) compared to those who had not (*n* = 153). The median age of the prior BCMA-TT cohort was 66 years, 66% were male, 19% had an Eastern Cooperative Oncology Group performance status (ECOG PS) ≥ 2, 27% had R-ISS stage III disease prior to infusion, 50% had extramedullary disease, and 36% had high-risk cytogenetics as defined by t(4;14), t(14;16), or del(17p). Patients in both cohorts were heavily pretreated, and the prior BCMA-TT cohort patients were more likely to have penta-refractory disease (62% versus 37%; *p* = 0.002) and had received a greater median number of prior therapies (nine versus six; *p* < 0.001) compared to the cohort who had not received a prior BCMA-TT.

**Table 1:** Baseline characteristics of patients infused with ide-cel.

Characteristic	SOC Ide-cel with prior BCMA-TT (*N* = 50)	SOC Ide-cel without prior BCMA-TT (*N* = 153)
Median age (range)	66 (43–79)	63 (36–83)
Male gender, *n* (%)	33 (66)	89 (58)
Race/Ethnicity, *n* (%)		
White	33 (66)	117 (76)
Black, Hispanic, Asian or other	17 (34)	36 (24)
ECOG PS, *n* (%)		
0–1	39 (81)	123 (83)
2–4	9 (19)	25 (17)
R-ISS stage, *n* (%)		
I	4 (11)	28 (24)
II	23 (62)	57 (48)
III	10 (27)	33 (28)
Extramedullary disease, *n* (%)	25 (50)	85 (56)
High tumor burden^a^, *n* (%)	13 (30)	42 (29)
High-risk cytogenetics, *n* (%)		
Any high-risk^b^	17 (36)	42 (31)
del(17p)	10 (21)	30 (22)
t(4;14)^*^	11 (23)	10 (8)
t(14;16)	1 (2)	6 (5)
Bridging therapy, *n* (%)	43 (86)	113 (74)
Median prior lines of therapy (range)^*^	9 (4–18)	6 (4–19)
Prior autologous SCT, *n* (%)	44 (88)	128 (84)
Prior allogeneic SCT, *n* (%)	2 (4)	10 (6.5)
Refractory status, *n* (%)		
Triple-class refractory^c^	45 (90)	125 (82)
Penta-drug refractory^*d^	31 (62)	57 (37)
Ide-cel dose (×10^6^), median (range)	403.3 (154.1–454.0)	406.7 (253.4–456.4)
Ide-cel dose (×10^6^), mean (SD)	392.4 (55.1)	399.3 (43.3)
<400 × 10^6^, *n* (%)	23 (46.0)	64 (42.1)
≥400 × 10^6^, *n* (%)	27 (54.0)	88 (57.9)

*SOC* standard of care, *BCMA-TT* BCMA targeted therapy, *ECOG PS* Eastern Cooperative Oncology Group Performance Status, *R-ISS* Revised International Staging System, SCT stem cell transplantation.

*Statistically significant difference between cohorts, *p* < 0.05.

^a^High tumor burden defined by ≥50% clonal plasma cells in pre-lymphodepletion chemotherapy bone marrow biopsy.

^b^As defined by the presence of del(17p), t(4;14) or t(14;16).

^c^Defined as refractory to ≥1 immunomodulatory drug, ≥1 proteasome inhibitor, and ≥1 anti-CD38 monoclonal antibody.

^d^Penta-refractory defined as refractory to ≥2 immunomodulatory drugs, ≥2 proteasome inhibitors, and ≥1 anti-CD38 monoclonal antibody.

Regarding the specific type of BCMA-TT received before ide-cel, 38 (76%) patients received an ADC, of which the vast majority was commercially available belantamab mafodotin. Seven (14%) had received a prior bispecific antibody and five (10%) had gotten a prior CAR T, with all prior bispecifics and CAR therapies received on a clinical trial. Amongst the prior CAR T subgroup, two patients had received ide-cel as their prior CAR construct on early phase trials, two had received an allogeneic CAR, and one received an autologous CAR with a non-viral transduction method. Further details about the clinical course for the five prior CAR T patients can be viewed in Supplemental Table 1. The median duration of exposure to the prior BCMA-TT was 30 days (range 1–370), median time from last exposure to the first BCMA-TT to leukapheresis was 160 days (range 1–1066), and median time to ide-cel infusion from last exposure was 202.5 days (range 16–1118). Twenty patients (40%) received commercial ide-cel within six months of the last exposure to their first BCMA-TT, and nine of these patients (18%) received ide-cel within three months. The ORR to the prior BCMA-TT was 21% overall, with six (17%) responding to a prior ADC, zero (0%) responding to the prior bispecific, and four (80%) attaining a response to the prior anti-BCMA CAR T (Supplemental Table 2). Notably 5/7 (71%) of the patients who received a prior bispecific antibody had received a suboptimal dose, meaning a dose level lower than what was chosen for dose expansion on the respective clinical trial.

### Safety

Adverse events are summarized for both cohorts in Table 2. The median duration of hospitalization for the prior BCMA-TT cohort was ten days (range 6–66), and four patients (8.0%) required intensive care unit level of care during their inpatient stay. The overall incidence of CRS for the prior BCMA-TT cohort was 80%. Only one patient (2%) in this group experienced grade 3 CRS, and no patients experienced a grade ≥ 4 CRS event. Median time to onset of maximum grade CRS was one day in both cohorts. The overall incidence of ICANS was 17% in the prior BCMA-TT cohort, with 4.3% of patients having grade 1 and 2 ICANS respectively, 2.1% grade 3, 4.3% grade 4, and one patient (2.1%) experiencing grade 5 ICANS. Rates of tocilizumab, anakinra, and glucocorticoid use did not differ significantly between cohorts.

**Table 2:** Adverse events by cohort.

Adverse event	SOC Ide-cel with prior BCMA-TT *N* (%)	SOC Ide-cel without prior BCMA-TT *N* (%)
Cytokine release syndrome (CRS)	*N* = 50	*N* = 153
Any grade	40 (80)	132 (86)
Grade 0	10 (20)	21 (14)
Grade 1	33 (66)	99 (65)
Grade 2	6 (12)	28 (18)
Grade 3	1 (2)	2 (1.3)
Grade 4	0 (0)	1 (0.7)
Grade 5	0 (0)	2 (1.3)
Grade ≥ 3	1 (2)	5 (3.3)

Immune-effector cell-associated neurotoxicity syndrome (ICANS)	*N* = 47	*N* = 144
Any Grade	8 (17)	31 (22)
Grade 0	39 (83)	113 (78)
Grade 1	2 (4.3)	14 (9.7)
Grade 2	2 (4.3)	7 (4.9)
Grade 3	1 (2.1)	6 (4.2)
Grade 4	2 (4.3)	4 (2.8)
Grade 5	1 (2.1)	0 (0)
Grade ≥ 3	4 (8.5)	10 (6.9)

Hematologic toxicity in first 30 days post ide-cel infusion	*N* = 50	*N* = 152
Neutropenia		
Any Grade	44 (88)	141 (93)
Grade ≥ 3	38 (76)	118 (78)
Anemia		
Any Grade	50 (100)	152 (100)
Grade ≥ 3	19 (38)	52 (35)
Thrombocytopenia		
Any Grade	49 (98)	144 (95)
Grade ≥ 3	32 (64)	87 (57)
Grade 4^a^	23 (46)	48 (32)

Supportive care for cytopenias	Variable *N*^c^	Variable *N*^c^
G-CSF use	37 (82)	115 (77)
TPO mimetic use^b^	12 (27)	17 (12)
Stem cell boost	4 (8.2)	7 (4.7)
**Infection**	11 (34)	34 (39)
**Tocilizumab use**	30 (63)	108 (73)
**Glucocorticoid use**	12 (24)	44 (29)
**Anakinra use**	3 (6)	7 (4.6)

*SOC* standard of care, *BCMA-TT BCMA* targeted therapy, *G-CSF* granulocyte colony-stimulating factor, *TPO* thrombopoietin.

^a^Difference between the two cohorts trended for significance, *p* = 0.064.

^b^Difference between the two cohorts was statistically significant, *p* = 0.013.

^c^Variable number of patients evaluable due to missing data for some patients in each category for the following variables: supportive care for cytopenias, infection, tocilizumab use, glucocorticoid use, and anakinra use.

Within the first 30 days after ide-cel infusion, grade ≥ 3 anemia, neutropenia, and thrombocytopenia occurred in 38%, 76%, and 64% of the patients in the prior BCMA-TT cohort respectively. There was a trend towards higher rates of grade 4 thrombocytopenia in the prior BCMA-TT cohort (46% versus 32%; *p* = 0.064), which translated to a higher rate of thrombopoietin mimetic use (27% versus 12%; *p* = 0.013) for the prior BCMA-TT group. The rate of documented infection was similar between cohorts at 34% for the prior BCMA-TT cohort and 39% for those with no prior BCMA-TT.

At data cutoff, 14 patients who had received commercial ide-cel died in the prior BCMA-TT cohort (10 from progressive myeloma; 1 toxicity/ICANS, 1 COVID-19, and 2 cardiac/unrelated non-relapse mortality; Supplemental Table 3). The increase in the incidence of death observed in the prior BCMA-TT cohort (28% versus 13%; *p* = 0.014) was driven by a significant increase in death related to myeloma disease progression for this cohort (20% versus 8%; *p* = 0.016). There was no significant increase in the incidence of death due to non-relapse mortality in the prior BCMA-TT cohort (8% versus 3%; *p* = 0.16).

### Treatment response

**Fig. 1:**
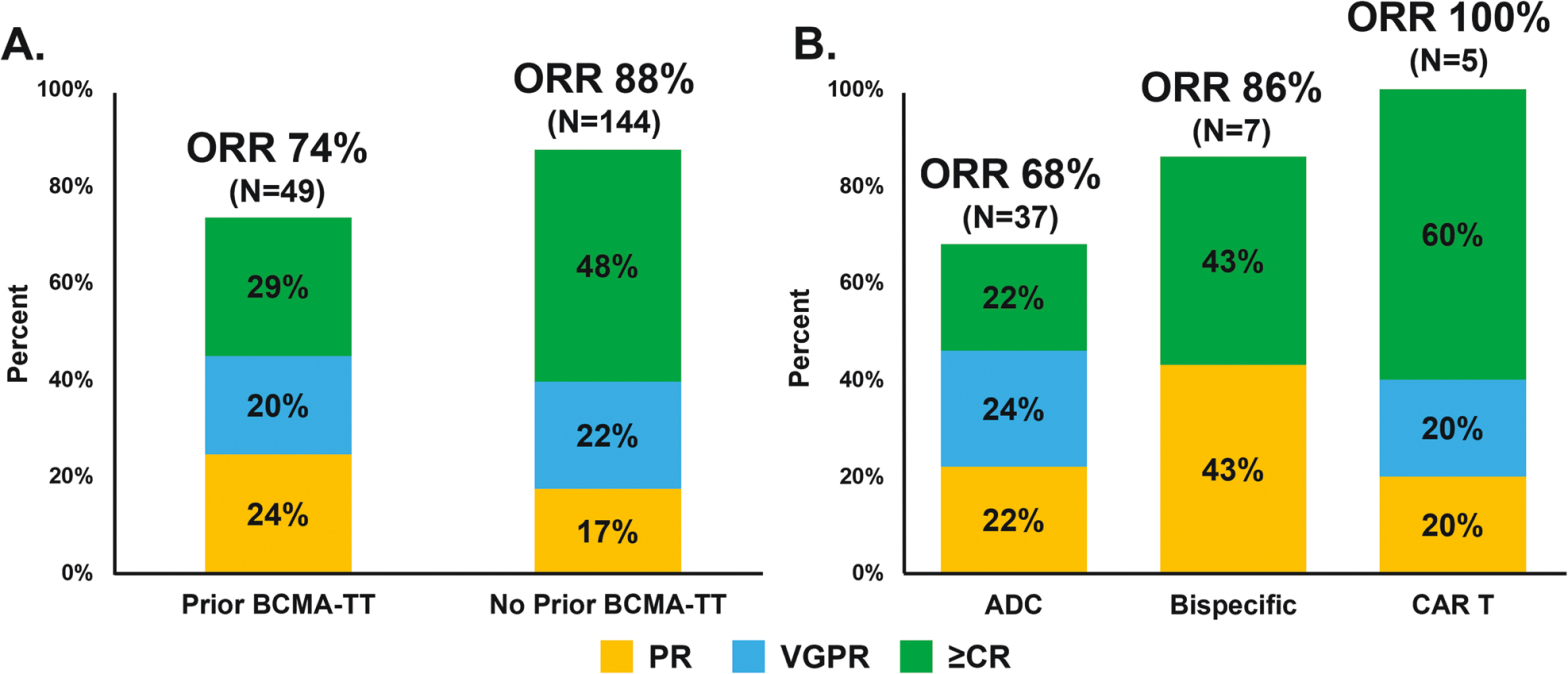
Response rates to ide-cel. Overall response rate and depth of response outcomes for the prior BCMA-TT cohort compared to the no prior BCMA-TT cohort (**A**), and stratified by the specific type of prior BCMA-TT (**B**). ORR overall response rate, CR complete response, VGPR very good partial response, PR partial response.

Best ORR to commercial ide-cel was evaluable for 49 patients in the prior BCMA-TT cohort and 144 patients for the no prior BCMA-TT group. Patients in the prior BCMA-TT cohort had a lower ORR (74% versus 88%; *p* = 0.021) and best response of ≥CR (29% versus 48%; *p* = 0.018) compared to the cohort who had not received a prior BCMA-TT (Fig. 1A). Figure 1B highlights the response rates to ide-cel by the specific type of prior BCMA-TT, which were best for the prior CAR T subgroup (ORR 100%; ≥CR 60%), followed by the prior bispecific subgroup (ORR 86%; ≥CR 43%) and ADC-exposed subgroup (ORR 68%; ≥CR 22%). When analyzing the prior BCMA-TT cohort by responders versus non-responders, patients who responded to ide-cel had a shorter median duration of exposure to their prior BCMA-TT (23 versus 63 days; *p* = 0.025), a longer amount of time from their last BCMA-TT exposure to apheresis (170 versus 84 days; *p* = 0.017), and a longer time to ide-cel infusion (209 versus 128 days; *p* = 0.052) compared to non-responders (Table 3). While patients receiving ide-cel within six months of their last BCMA-TT exposure had a numerically lower ORR (60% versus 83%; *p* = 0.076) and best response of CR or better (20% versus 35%; *p* = 0.48) compared to the patients receiving ide-cel >6 months after their last BCMA-TT exposure, this did not reach statistical significance. The nine patients receiving ide-cel within three months of their last BCMA-TT also had a numerically lower ORR (67% versus 75%; *p* = 0.61) compared to those receiving ide-cel >3 months after the last exposure.

**Table 3:** Selected variables for ide-cel responders compared to non-responders in the prior BCMA-TT cohort.

Variable	Responders (*N* = 36)	Non-responders (*N* = 13)	*P*
Duration of therapy with prior BCMA-TT in days, median (range)^a^	23 (1–208)	63 (1–370)	0.025
Time from last BCMA-TT to apheresis in days, median (range)	169.5 (30–1066)	84 (1–286)	0.017
Time from last BCMA-TT to ide-cel infusion in days, median (range)	209 (16–1118)	128 (32–362)	0.052
Ide-cel cell dose (×10^**6**^), mean (SD)	392.3 (58.9)	397.7 (43.7)	0.95
Received systemic therapy between last BCMA-TT and apheresis, *n* (%)	28 (78%)	9 (69%)	0.539

^a^Note that prior anti-BCMA CAR T was recorded as 1 day for duration of prior BCMA-TT.

The median DOR was shorter in the prior BCMA-TT cohort compared to the no prior BCMA-TT cohort (7.4 versus 9.6 months; *p* = 0.03). The median DOR by specific type of prior BCMA-TT were 7.4 months, 2.8 months, and not reached for prior ADC, bispecific, and CAR T respectively (Supplemental Table 4). Patients who responded to ide-cel after receiving a prior bispecific had a lower median DOR compared to the patients responding to ide-cel after a different type of prior BCMA-TT (2.8 months versus 8.9 months; *p* = 0.053).

A univariate analysis of the patients who had received a prior BCMA-TT (Supplemental Table 5) demonstrated that having penta-refractory disease was associated with a lower likelihood of attaining an ORR to ide-cel (*p* = 0.053). Having attained a response of ≥PR to the initial BCMA-TT was not associated with an increased likelihood of attaining a response of ≥PR or ≥CR to commercial ide-cel. In a multivariable analysis including all patients who received ide-cel (Table 4), prior BCMA-TT was associated with a lower likelihood of attaining a best response of ≥CR (OR 0.29; 95% CI 0.13–0.66; *p* = 0.003).

**Table 4:** Multivariable analysis for the association of prior BCMA-TT and selected patient and disease characteristics with best ORR, best response ≥ CR, PFS, and OS.

	Best response ≥CR (*N* = 168)			Best ORR (*N* = 168)			PFS (*N* = 176)			Overall survival (*N* = 176)		
Characteristic	N (Event N)	OR (95% CI)	*P*	N (Event N)	OR (95% CI)	*P*	N (Event N)	HR (95% CI)	*P*	N (Event N)	HR (95% CI)	*P*
**Prior BCMA-TT**												
No	124 (62)	1.00 (Ref)		124 (109)	1.00 (Ref)		131 (44)	1.00 (Ref)		131 (17)	1.00 (Ref)	
Yes	44 (12)	0.29 (0.13–0.66)	**0.003**	44 (33)	0.49 (0.19–1.23)	0.13	45 (23)	2.91 (1.68–5.04)	**<0.0001**	45 (12)	3.44 (1.45–8.14)	**0.005**
**High-risk FISH**^**a,b**^												
No	115 (53)	1.00 (Ref)		115 (96)	1.00 (Ref)		118 (38)	1.00 (Ref)		118 (15)	n/a	
Yes	53 (21)	0.83 (0.41–1.67)	0.59	53 (46)	1.13 (0.42–3.07)	0.81	58 (29)	2.55 (1.51–4.30)	**<0.0001**	58 (14)	n/a	n/a
**EM Disease**												
No	91 (37)	1.00 (Ref)		91 (80)	1.00 (Ref)		91 (26)	1.00 (Ref)		91 (11)	1.00 (Ref)	
Yes	77 (37)	1.70 (0.87–3.33)	0.12	77 (62)	0.67 (0.27–1.68)	0.39	85 (41)	1.58 (0.95–2.65)	0.079	85 (18)	1.49 (0.65–3.40)	0.34
**ECOG PS at LD**												
0-1	140 (63)	1.00 (Ref)		140 (120)	1.00 (Ref)		143 (47)	1.00 (Ref)		143 (19)	1.00 (Ref)	
2-4	28 (11)	0.78 (0.32–1.91)	0.59	28 (22)	0.84 (0.28–2.55)	0.76	33 (20)	2.73 (1.53–4.87)	**0.001**	33 (10)	3.79 (1.60–8.99)	**0.002**
**Penta-refractory**												
No	97 (41)	1.00 (Ref)		97 (87)	1.00 (Ref)		102 (38)	1.00 (Ref)		102 (18)	1.00 (Ref)	
Yes	71 (33)	1.64 (0.84–3.24)	0.15	71 (55)	0.48 (0.19–1.18)	0.11	74 (29)	0.74 (0.44–1.24)	0.26	74 (11)	0.55 (0.24–1.26)	0.16
**Patient age at infusion**												
<65 years	88 (35)	1.00 (Ref)		88 (72)	1.00 (Ref)		92 (42)	1.00 (Ref)		92 (15)	1.00 (Ref)	
≥65 years	80 (39)	1.86 (0.95–3.64)	0.069	80 (70)	1.32 (0.52–3.33)	0.56	84 (25)	0.42 (0.24–0.72)	**0.002**	84 (14)	0.61 (0.27–1.39)	0.24

Bold values highlight where *P*-value < 0.05.

*CR* complete response, *ORR* overall response rate, *PFS* progression-free survival, *OR* odds ratio, *CI* confidence interval, *P* P-value by chi-square test, *HR* hazard ratio, *BCMA-TT* BCMA-targeted therapy, *FISH* fluorescence in situ hybridization, *EM* extramedullary, *ECOG PS* Eastern Cooperative Oncology Group Performance Status, *LD* lymphodepletion.

^a^High-risk FISH includes del(17p), t(4;14), and t(14;16).

^b^In the model for OS, high-risk FISH violated the proportional hazards assumption and this variable was included as a strata term in the model.

### Survival outcomes

**Fig. 2:**
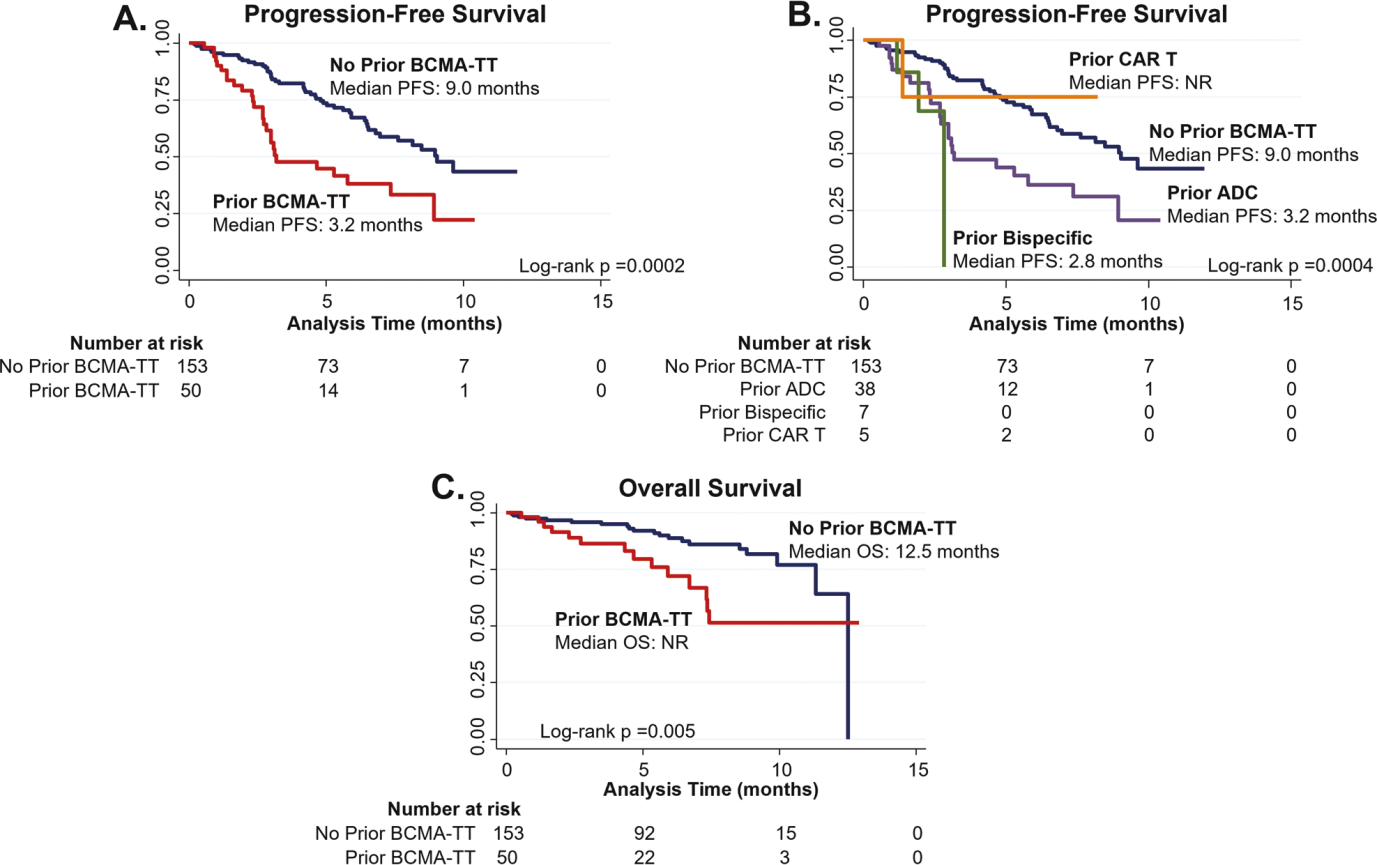
Progression-free survival and overall survival. Kaplan–Meier curves demonstrating PFS in the prior BCMA-TT cohort compared to the no prior BCMA-TT cohort (**A**), PFS stratified by the specific type of prior BCMA-TT (**B**), and overall survival in the prior BCMA-TT cohort compared to the no prior BCMA-TT cohort (**C**).

The median duration of follow up was 4.5 months for the prior BCMA-TT cohort and six months for the cohort without prior BCMA-TT exposure. Patients who had received a prior BCMA-TT had a lower median PFS compared to patients who had received no prior BCMA-TT (3.2 months versus 9.0 months; *p* = 0.0002). The median PFS by specific type of prior BCMA-TT were not reached, 3.2 months, and 2.8 months for the prior CAR T, prior ADC, and prior bispecific groups respectively (Fig. 2A, B). Median OS analyses were limited by the short duration of follow-up (Fig. 2C). The 3-month and 6-month OS rates were 87% and 72% for the prior BCMA-TT cohort, compared to 96% and 89% respectively in the no prior BCMA-TT cohort.

Multivariable analysis demonstrated that treatment with a prior BCMA-TT was an independent predictor for both inferior PFS (HR, 2.91; 95% CI, 1.68–5.04; *p* < 0.0001) and inferior OS (HR, 3.44; 95% CI 1.45–8.14; *p* = 0.005). In addition, ECOG PS ≥ 2 was predictive of both inferior PFS and OS, while high-risk cytogenetics and age < 65 were associated with worse PFS (Table 4).

## Discussion

This consortium previously reported on a cohort of 159 patients with RRMM who received ide-cel in the real-world setting, which demonstrated comparable safety and efficacy outcomes compared to those observed on the KarMMa trial despite 75% of patients not meeting the eligibility criteria [13]. The patients represented in this study include an extended duration of follow up for the first 159 patients infused, as well as 44 additional patients who received ide-cel since the last data cutoff. To our knowledge, this analysis of patients who received commercial ide-cel after a prior BCMA-TT (*n* = 50) represents the largest cohort described for a specific anti-BCMA therapy after prior exposure to another BCMA-TT. The prior BCMA-TT cohort consisted of heavily pre-treated patients (median nine prior lines of therapy, 62% penta-drug refractory) with aggressive disease characteristics (19% ECOG PS ≥ 2, 50% extramedullary disease, 36% high-risk cytogenetics). Ide-cel manufacturing and infusion were feasible in this real-world cohort of patients who had received prior anti-BCMA therapy given the comparable rates of manufacture failure (1.8% versus 2.7%) and ide-cel infusion in the intention-to-treat population (91% versus 92%) when compared to the cohort who had not received a prior BCMA-TT.

Despite a low ORR of 21% to the prior BCMA-TT, treatment with commercial ide-cel yielded an impressive ORR of 74%. Although the ORR was significantly lower than that observed in the no prior BCMA-TT cohort (74% versus 88%), the ORR observed with ide-cel compares favorably with those reported for other BCMA-targeted therapies in this setting. Cohort C of the prospective CARTITUDE-2 study consisted of 20 patients infused with cilta-cel after prior exposure to a non-cellular BCMA-TT (13 ADC, 7 bispecific). Cilta-cel treatment resulted in an ORR of 60%, median DOR 11.5 months, and median PFS of 9.1 months [11]. Preliminary data from cohort C of the MajesTEC-1 study included 40 patients treated with teclistamab after exposure to a prior BCMA-TT (29 ADC, 15 CAR T) and noted an ORR of 52.5% [9]. The MagnetisMM-1 study of the bispecific antibody elranatamab included 13 patients who had received a prior BCMA-TT (8 ADC, 9 CAR T), and preliminary results noted an ORR of 54% [10]. While ORR and depth of response outcomes were favorable in our prior BCMA-TT real-world cohort of patients treated with ide-cel, it should be noted that the one patient who died prior to first response assessment was not included in the response analysis, and responses were graded by investigators at each institution without mandated confirmatory testing/imaging due to the retrospective nature of the study.

Response rates to ide-cel in our prior BCMA-TT cohort also compare favorably to the current commercially available non-BCMA therapies for patients with triple-class refractory myeloma who have also received a prior anti-BCMA therapy. Preliminary data from the STOMP study included 11 patients who had progressed after a prior BCMA-TT and were treated with heterogeneous selinexor-containing regimens. The ORR was 64% for this trial population, but a real-world study of patients with disease progression after BCMA CAR T noted lower rates of response to salvage selinexor-based therapy [16, 17]. Given the limited novel treatment options available for such patients, clinicians often consider retreatment with doublet or triplet regimens consisting of previously received agents for patients with disease progression after anti-BCMA therapy. However, the observed ORR with this strategy in the real-world setting was 28% in patients with progression after BCMA CAR T [17]. Therapies targeting G Protein-Coupled Receptor Family C Group 5 Member D (GPRC5D) such as the bispecific antibody talquetamab and several anti-GPRC5D CAR T therapies have demonstrated high response rates post-BCMA therapy, but access to GPRC5D-targeted agents is currently limited to clinical trials [18–21]. Other investigational agents that have demonstrated modest efficacy in BCMA-exposed RRMM include cevostamab, mezigdomide plus dexamethasone, and modakafusp alfa [22–24].

Although the ORR to ide-cel for patients in our prior BCMA-TT cohort was relatively high, this group of patients had a significantly lower ORR (74% versus 88%), rate of ≥CR (29% versus 48%), median DOR (7.4 versus 9.6 months), and median PFS (3.2 versus 9.0 months) compared to our real-world cohort without prior BCMA-TT exposure. Patients in the prior BCMA-TT cohort had received a greater median number of prior lines of therapy and were more likely to have penta-refractory disease, which may partially explain the observed worse efficacy outcomes. However, multivariable analysis demonstrated that treatment with a prior anti-BCMA therapy was an independent predictor of worse PFS and OS, as well as a lower likelihood of having a best response of ≥CR. Future prospective studies investigating anti-BCMA CAR T in patients with prior BCMA-TT exposure should give consideration to combination strategies, consolidation approaches, or maintenance therapies in order to extend the duration of clinical benefit. As standard-of-care anti-BCMA CAR T therapies remain a limited resource due to manufacturing slot allocation, it is important to consider potential prognostic and predictive factors such as the specific type and timing of prior BCMA-TT exposure in patient selection for commercial CAR T.

The subgroup of patients who had received an anti-BCMA CAR T prior to ide-cel had the best outcomes in terms of ORR (100%), median DOR, PFS, and OS (all not reached) compared to patients who had received a prior ADC or bispecific. While these results should be interpreted with caution given the small sample size (*n* = 5), our study indicates that sequential anti-BCMA CAR T can be an efficacious strategy and prior anti-BCMA CAR T treatment should not be an exclusion criterion for future prospective studies of investigational anti-BCMA CAR T-cell therapies. Several ongoing studies have demonstrated high response rates to investigational GPRC5D-targeted CAR T-cell therapies after prior anti-BCMA CAR T, further validating the potential utility of sequential cellular therapies in RRMM [19–21].

In the KarMMa study, 28 patients were retreated with ide-cel at a higher dose level after disease progression from their first CAR T infusion. The ORR for the retreated patients was low at 21% (0% ≥ CR), with DOR ranging from 1.9 to 6.8 months and a median PFS of 1.0 months [5] Both patients in our study who had received ide-cel as their prior anti-BCMA CAR T attained deep responses ≥ CR when retreated with commercial ide-cel. These patients both received commercial ide-cel over three years after their first ide-cel infusion on trial, and both also received several interval therapies between the two anti-BCMA CAR T therapies (Supplemental Table 1). Compared to the KarMMa trial patients who received ide-cel retreatment as the next line of therapy after progression from the first infusion, the longer time to second infusion and use of anti-myeloma therapies with alternative mechanisms of action between the two CAR T infusions may explain the improved outcomes seen with retreatment in our real-world analysis.

While the ORR to ide-cel for the patients who had received a prior anti-BCMA bispecific was high at 86%, these patients numerically had the lowest median DOR, PFS, and OS compared to those receiving a prior ADC or CAR T. The median DOR was significantly lower for patients receiving a prior bispecific compared to an alternative type of BCMA-TT (2.8 versus 8.9 months). This should be interpreted with caution due to small sample size (*n* = 7). Also, the response rate to the prior bispecific was 0% and thus not representative of the expected ~60% ORR seen on clinical trials for several BCMA-targeted bispecific antibodies [7, 10, 25]. The poor ORR seen with the prior bispecific may have reflected more treatment-refractory disease biology, but it is also important to note that 71% of patients in this subgroup received a dose on the respective phase 1/2 trial that was lower than the dose later chosen for dose-expansion. Endogenous T-cell exhaustion from continuous antigenic pressure may be a potential explanation for the poor durability of response observed with ide-cel for patients who had received a prior bispecific antibody. Preclinically, continuous bispecific antibody exposure has been shown to induce T-cell exhaustion over time and this may be ameliorated by treatment-free intervals [26]. In the CARTITUDE-2 cohort C analysis, patients receiving cilta-cel after a prior bispecific had a numerically lower median DOR and PFS compared to those who had received a prior ADC (8.2 versus 11.5 months and 5.3 versus 9.5 months respectively) [11]. In a dedicated group of patients who had received a T-cell redirecting therapy prior to the GPRC5D-targeted bispecific talquetamab, patients who had received a prior CAR T had a higher ORR than those who had received a prior bispecific (72% versus 44%) [18].

While the aggregate data regarding sequencing of T-cell redirecting therapies seems to support the use of CAR T prior to bispecific antibodies, this strategy may not always be feasible due to access limitations related to CAR T manufacturing and the need for administration at highly specialized centers. For patients being considered for anti-BCMA CAR T after a prior BCMA-TT, the timing between sequential BCMA-TT may be a potential predictor of response. Patients in our study who responded to ide-cel had a significantly shorter duration of exposure to their prior BCMA-TT, and a significantly longer amount of time from their last BCMA-TT exposure to both apheresis and ide-cel infusion when compared to non-responders. Similar findings related to prior BCMA-TT timing were observed with cilta-cel in the CARTITUDE-2 cohort C analysis when stratifying by responders versus non-responders [11]. Although these results may be confounded by more indolent myeloma disease biology allowing for more time between anti-BCMA therapies, these data suggest that using alternative therapies with different mechanisms of action may be a reasonable strategy to extend the duration of time between BCMA-targeted therapies.

Biallelic BCMA loss has been described as a resistance mechanism, albeit relatively uncommon [5, 27]. A recent serial analysis of bone marrow aspirates for patients receiving a BCMA-TT revealed distinct genomic mechanisms of BCMA antigen escape mediated resistance. These mechanisms include focal biallelic loss of BCMA while on therapy, a pre-existing subclone with biallelic BCMA loss followed by clonal expansion on anti-BCMA therapy leading to disease progression, and monoallelic BCMA loss combined with either point mutations or inframe deletions in the other BCMA locus leading to functional BCMA loss [28]. Only ten of the 50 patients treated with ide-cel after prior BCMA-TT exposure in our study had testing for BCMA-expression performed on the pre-lymphodepletion bone marrow biopsy, all of which were positive for BCMA expression at varying levels of intensity. One hypothesis for the inferior DOR and PFS outcomes seen in the prior BCMA-TT cohort is that the prior anti-BCMA therapy may have contributed to a greater rate of development of myeloma subclones with monoallelic or biallelic BCMA loss that then experienced clonal expansion amidst the therapeutic pressure of ide-cel treatment. Unfortunately, BCMA expression testing was not available after disease relapse on ide-cel due to the retrospective study design. Future prospective studies should consider assessment for such subclones prior to treatment and further BCMA profiling at the time of disease relapse to better understand these resistance mechanisms.

Strengths of this study include analysis of the largest multi-institutional cohort of patients receiving a specific anti-BCMA CAR T after exposure to another BCMA-TT, who would not have been eligible to receive ide-cel on the KarMMa trial. Limitations of our study include the retrospective design, small sample sizes for each individual type of prior BCMA-TT, response assessment per investigator discretion without mandating confirmatory testing or imaging, and limited duration of follow-up at time of data cutoff. Another limitation that may impact future generalizability is that the majority of our prior BCMA-TT cohort received belantamab mafodotin, which has since been withdrawn from commercial use.

In summary, treatment with commercial ide-cel after prior BCMA-TT resulted in a relatively high ORR, but significantly lower ORR, median DOR, and median PFS compared to patients not receiving a prior BCMA-TT. The timing of ide-cel infusion in relation to the last exposure to a prior BCMA-TT may be predictive of the likelihood of response, and the small subgroup of patients who received sequential anti-BCMA CAR T had the best response and PFS outcomes.

### Supplementary information

Supplemental Material

## Data Availability

Data will be made available upon reasonable request. Data requestors will need to sign a data access agreement and submit a proposal to Dr. Krina Patel to be reviewed and approved by the U.S. Multiple Myeloma Immunotherapy Consortium Steering Committee.
