# Supplementary material for: Real-world experience of patients with multiple myeloma receiving ide-cel after a prior BCMA-targeted therapy
Source: Blood Cancer J. 2023 Aug 9;13(1):117. doi: 10.1038/s41408-023-00886-8 (PMC10412575; doi:10.1038/s41408-023-00886-8)
Supplement: Supplementary file 1 — Supplemental Material [file 41408_2023_886_MOESM1_ESM.pdf]

Supplementary Material: Real-world experience of patients with multiple myeloma receiving ide-cel after a prior BCMA-targeted therapy

Table of Contents

Appendix 1. Supplementary Methods 2

Supplemental Figure 1. Study flow chart of the prior BCMA-TT cohort 3

Supplemental Figure 2. Study flow chart of the no prior BCMA-TT cohort 4

Supplemental Table 1. Clinical course highlights for prior anti-BCMA CAR T subgroup 5

Supplemental Table 2. Best response to prior BCMA-targeted therapies 7

Supplemental Table 3. Causes of death in each cohort 8

Supplemental Table 4. Duration of response outcomes 9

Supplemental Table 5. Univariate analysis for characteristics associated with toxicity and response in the prior BCMA-TT cohort only 10

Appendix 1. Supplementary Methods

A uniform data collection form with embedded data dictionary was provided to all participating centers by the coordinating center, along with an example on guidelines for data collection. All sites returned data to the coordinating center. A quality control check was done by the coordinating center, and queries were issued for missing data or data that did not follow the format specified in the data collection form. Overall survival was calculated as time between the date of infusion and date of death from any cause or last contact, and progression-free survival was calculated as time between the date of infusion and date of progression, death, or last contact. DOR was calculated as time between the date of infusion and date of progression, death, or last contact for patients who achieved a partial response or better. We performed multivariable logistic regression to examine the association of a priori selected patient characteristics (prior B-cell maturation antigen–targeted therapy [yes, no], high-risk cytogenetics [yes, no], extramedullary disease [yes, no], Eastern Cooperative Oncology Group performance status [0-1, 2-4], penta-refractory disease [yes, no], and patient age at infusion [< 65 years versus ≥ 65 years].

Supplemental Figure 1. Study flow chart of the prior BCMA-TT cohort

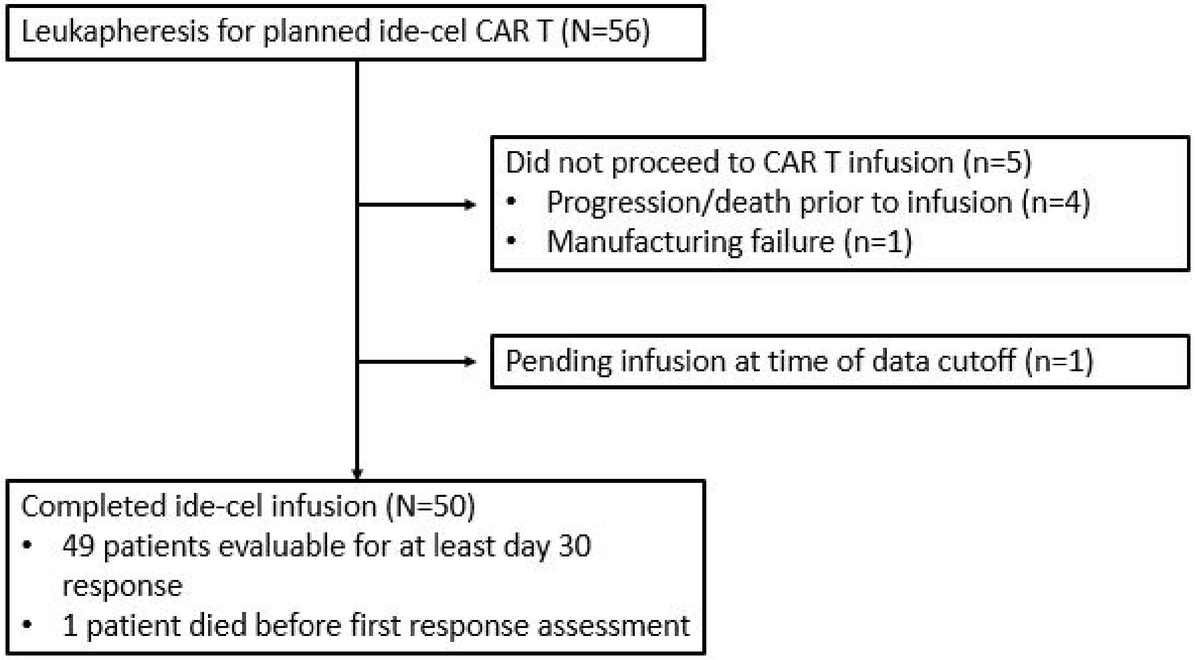

Supplemental Figure 2. Study flow chart of the no prior BCMA-TT cohort

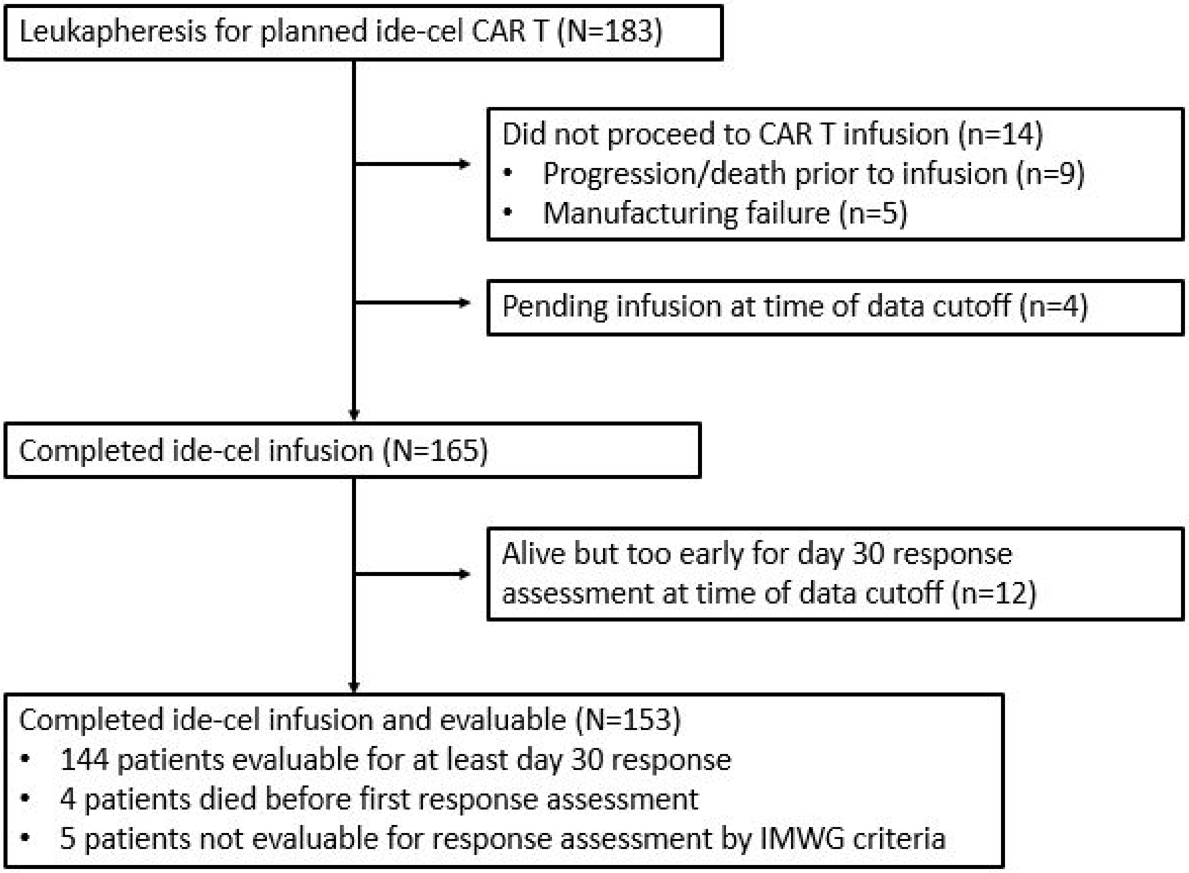

Supplemental Table 1. Clinical course highlights for prior anti-BCMA CAR T subgroup

Patient Characteristics at time of SOC ide-cel	First anti-BCMA CAR T-cell therapy (Best response)	Duration between first CAR T and SOC ide-cel (List of interval therapies)	BCMA testing prior to ide-cel	SOC ide-cel course and efficacy
69 yo M; 5 prior LOT including autoSCT; penta-exposed; EM disease	Ide-cel construct on early phase trial (PR)	37.3 months (XRT; K-Seli-dex; DCEP; Dara-Pom-Cy-dex bridging)	Not done	G2 CRS; no ICANS; MRD-negative sCR with ongoing response at data cutoff
71 yo M; 18 prior LOT including autoSCT; penta-refractory; HR-FISH†	Ide-cel construct on early phase trial (sCR, PD at 30.9 months)	36.1 months (NKTR-255 on trial; venetoclax-bortezomib-dex; K-Pano-dex; modified hyper-CBAD bridging)	Yes, 3+ by IHC	G1 CRS, no ICANS, RSV LRTI; MRD-negative sCR with response ongoing at data cutoff, but noted to have t-MDS on day +30 BMBx
50 yo M; 6 prior LOT including autoSCT; penta-refractory; HR-FISH‡	Allogeneic CAR (CR)	7.5 months (KCd bridging, unclear if any given prior to apheresis)	Yes, bright (90%) by IHC	No CRS or ICANS; Shiga like E. coli gastroenteritis; VGPR; PD noted at 1.4 months and death from myeloma progression at 4.4 months post-infusion
62 yo M; 8 prior LOT including autoSCT; penta-refractory; HR-FISH*	Allogeneic CAR (PR)	6.1 months (Dara-Seli-dex bridging, unclear if any given prior to apheresis)	Yes, bright (90%) by IHC	G1 CRS; no ICANS; CR with ongoing response at data cutoff
50 yo M; 7 prior LOT including autoSCT; triple-refractory and penta-exposed; EM disease	Autologous CAR with non-viral transduction method (SD)	20.3 months (K-dex both prior to apheresis and as bridging therapy)	Yes, dim positive by flow	G1 CRS; no ICANS; PR with ongoing response at data cutoff

SOC, standard of care; CAR, chimeric antigen receptor; LOT, lines of therapy; autoSCT, autologous stem cell transplant; EM, extramedullary; PR, partial response; XRT, radiation therapy; K, carfilzomib; Seli, selinexor; dex, dexamethasone; DCEP, chemotherapy with dexamethasone, cyclophosphamide, etoposide, and cisplatin; Dara, daratumumab; Pom, pomalidomide; Cy; cyclophosphamide; G, grade; CRS, cytokine release syndrome; ICANS, immune-effector cell-associated neurotoxicity syndrome; MRD, minimal residual disease; sCR, stringent complete response; HR-FISH, high-risk fluorescence in-situ hybridization; PD, progression of disease; Pano, panobinostat; CBAD, chemotherapy with hyper-fractionated cyclophosphamide, bortezomib, doxorubicin, and dexamethasone; IHC, immunohistochemistry; RSV, respiratory syncytial virus; LRTI, lower respiratory tract infection; t-MDS, treatment-related myelodysplastic syndrome; BMBx, bone marrow aspiration and biopsy; KCd, carfilzomib, cyclophosphamide, dexamethasone; VGPR, very good partial response; SD, stable disease

† Patient noted to have t(4;14) and t(14;16) on bone marrow biopsy prior to apheresis for ide-cel

‡ Patient noted to have deletion 17p and t(4;14) on bone marrow biopsy prior to apheresis for ide-cel
* Patient noted to have deletion 17p on bone marrow biopsy prior to apheresis for ide-cel

Supplemental Table 2. Best response to prior BCMA-targeted therapies

Response outcome	N (%)
**ORR to prior BCMA-TT**	
All (N = 48)	10 (21%)
ADC (N = 36)*	6 (17%)
Bispecific (N = 7)	0 (0%)
CAR T (N = 5)	4 (80%)
**Depth of response to any prior BCMA-TT (N = 48)**	
≥ CR	3 (6%)
VGPR	3 (6%)
PR	4 (8%)
SD/MR	14 (29%)
PD	24 (50%)
**Depth of response to prior CAR T (N = 5)**	
≥ CR	2 (40%)
VGPR	0
PR	2 (40%)
SD/MR	1 (20%)
PD	0

ORR, overall response rate; ADC, antibody-drug conjugate; CAR T, chimeric antigen receptor T-cell; CR, complete response; VGPR, very good partial response; PR, partial response; SD, stable disease; MR, minimal response; PD, progressive disease.
*Two patients treated with a prior ADC were not evaluable for response to their prior ADC therapy

Supplemental Table 3. Causes of death in each cohort

Cause of Death	Prior BCMA-TT (N = 50)	No prior BCMA-TT (N = 153)	*P*
**Total Deaths**	14 (28%)	20 (13%)	**0.014**
**Myeloma-related death**	10 (20%)	12 (8%)	**0.016**
**Non-relapse mortality**	4 (8%)	5 (3%)	0.158
Toxicity (CRS/ICANS)	1 (2%)	2 (1%)	N/A
COVID-19	1 (2%)	2 (1%)	N/A
Cardiac (unrelated)	2 (4%)	0	N/A
Non-specified non-relapse mortality	0	1 (0.7%)	N/A
**Unknown**	0	3	N/A

BCMA-TT, BCMA-targeted therapy; CRS, cytokine release syndrome; ICANS, immune-effector cell-associated neurotoxicity syndrome

Supplemental Table 4. Duration of response outcomes

Cohort	Median duration of response (months)	*P*	
No Prior BCMA-TT (N=126)	9.6	**0.03**	
Prior BCMA-TT (N=36)	7.4		
**Cohort by type of prior BCMA-TT**	**Median duration of response (months)**	**Median duration of response for other BCMA-TT (months)**	***P***
Prior ADC (n=25)	7.4	Not reached (n=11)	0.59
Prior Bispecific (n=6)	2.8	8.9 (n=30)	**0.05**
Prior CAR T (n=5)	Not reached	7.4 (n=31)	0.57

BCMA-TT, BCMA-targeted therapy; ADC, antibody-drug conjugate, CAR T, chimeric antigen receptor T-cell

Supplemental Table 5. Univariate analysis for characteristics associated with toxicity and response in the prior BCMA-TT cohort only

	CRS			ICANS			Best response ≥ CR			Best ORR		
**Characteristic**	**< G2 N (%)**	**≥ G2 N (%)**	***P***	**< G2 N (%)**	**≥ G2 N (%)**	***P***	**< CR N (%)**	**≥ CR N (%)**	***P***	**< PR N (%)**	**≥ PR N (%)**	***P***
**Age**	43 (86.0)	7 (14.0)		41 (87.2)	6 (12.8)		35 (71.4)	14 (28.6)		13 (26.5)	36 (73.5)	
< 65 years	17 (39.5)	5 (71.4)	0.22	19 (46.3)	2 (33.3)	0.68	15 (42.9)	7 (50.0)	0.76	6 (46.2)	16 (44.4)	1.00
≥ 65 years	26 (60.5)	2 (28.6)		22 (53.6)	4 (66.7)		20 (57.1)	7 (50.0)		7 (53.8)	20 (55.6)	
**Sex**	43 (86.0)	7 (14.0)		41 (87.2)	6 (12.8)		35 (71.4)	14 (28.6)		13 (26.5)	36 (73.5)	
Male	27 (62.8)	6 (85.7)	0.40	28 (68.3)	4 (66.7)	1.00	22 (62.9)	10 (71.4)	0.74	6 (46.2)	26 (72.2)	0.17
Female	16 (37.2)	1 (14.3)		13 (31.7)	2 (33.3)		13 (37.1)	4 (28.6)		7 (53.9)	10 (27.8)	
**EM disease**	43 (86.0)	7 (14.0)		41 (87.2)	6 (12.8)		35 (71.4)	14 (28.6)		13 (26.5)	36 (73.5)	
Yes	20 (46.5)	5 (71.4)	0.42	22 (53.7)	3 (50.0)	1.00	16 (45.7)	8 (57.1)	0.54	7 (53.9)	17 (47.2)	0.75
No	23 (53.5)	2 (28.6)		19 (46.3)	3 (50.0)		19 (54.3)	6 (42.9)		6 (46.1)	19 (52.8)	
**Disease status**	42 (85.7)	7 (14.3)		40 (87.0)	6 (13.0)		35 (71.4)	14 (28.6)		13 (27.1)	35 (72.9)	
Relapsed	10 (23.8)	4 (58.1)	*0.091*	11 (27.5)	2 (33.3)	1.00	9 (26.5)	4 (28.6)	1.00	2 (15.4)	11 (31.4)	0.47
Refractory	32 (76.2)	3 (42.9)		29 (72.5)	4 (66.7)		25 (73.5)	10 (71.4)		11 (84.6)	24 (68.6)	
**Plasma cell leukemia**	43 (86.0)	7 (14.0)		41 (87.2)	6 (12.8)		35 (71.4)	14 (28.6)		13 (26.5)	36 (73.5)	
Yes	1 (2.3)	0 (0.0)	1.00	1 (2.4)	0 (0.0)	1.00	1 (2.9)	0 (0.0)	1.00	1 (7.7)	0 (0.0)	0.27
No	42 (97.7)	7 (100.0)		40 (97.6)	6 (100.0)		34 (97.1)	14 (100.0)		12 (92.3)	36 (100.0)	
**Amyloidosis**	43 (86.0)	7 (14.0)		41 (87.2)	6 (12.8)		35 (71.4)	14 (28.6)		13 (26.5)	36 (73.5)	
Yes	0 (0.0)	0 (0.0)	n/a	0 (0.0)	0 (0.0)	n/a	0 (0.0)	0 (0.0)	n/a	0 (0.0)	0 (0.0)	n/a
No	43 (86.0)	7 (14.0)		41 (100.0)	6 (100.0)		35 (100.0)	14 (100.0)		13 (100.0)	36 (100.0)	
**ECOG PS**	41 (85.4)	7 (14.6)		39 (86.7)	6 (13.3)		34 (72.3)	13 (27.7)		12 (25.5)	35 (74.5)	
0-1	22 (80.5)	6 (85.7)	1.00	32 (82.1)	4 (66.7)	0.58	27 (79.4)	11 (84.6)	1.00	8 (66.7)	30 (85.7)	0.21

2-4	8 (19.5)	1 (14.3)		7 (17.9)	2 (33.3)		7 (20.6)	2 (15.4)		4 (33.3)	5 (14.3)	
**R-ISS Stage**	32 (86.5)	5 (13.5)		31 (88.6)	4 (11.4)		27 (75.0)	9 (25.0)		9 (25.0)	27 (75.0)	
I	4 (12.5)	0 (0.0)	1.00	4 (12.9)	0 (0.0)	0.74	1 (3.7)	3 (33.3)	*0.075*	1 (11.1)	3 (11.1)	1.00
II	19 (59.4)	4 (80.0)		20 (64.5)	2 (50.0)		19 (70.4)	4 (44.4)		6 (66.7)	17 (63.0)	
III	9 (29.1)	1 (20.0)		7 (22.6)	2 (50.0)		7 (25.9)	2 (22.2)		2 (22.2)	7 (25.9)	
**Any high-risk FISH**	40 (85.1)	7 (14.9)		39 (88.6)	5 (11.4)		33 (71.7)	13 (28.3)		12 (26.1)	34 (73.9)	
Yes	15 (37.5)	2 (28.6)	1.00	14 (35.9)	2 (40.0)	1.00	12 (36.4)	4 (30.8)	1.00	4 (33.3)	12 (35.3)	1.00
No	25 (62.5)	5 (71.4)		25 (64.1)	3 (60.0)		21 (63.6)	9 (69.2)		8 (66.7)	22 (64.7)	
**ADC as prior BCMA-TT**	43 (86.0)	7 (14.0)		41 (87.2)	6 (12.8)		35 (71.4)	14 (28.6)		13 (26.5)	36 (73.5)	
Yes	33 (76.7)	5 (71.4)	1.00	30 (73.2)	5 (83.3)	1.00	29 (82.9)	8 (57.1)	*0.076*	12 (92.3)	25 (69.4)	0.14
No	10 (23.3)	2 (28.6)		11 (26.8)	1 (16.7)		6 (17.1)	6 (42.9)		1 (7.7)	11 (30.6)	
**Bispecific as prior BCMA-TT**	43 (86.0)	7 (14.0)		41 (87.2)	6 (12.8)		35 (71.4)	14 (28.6)		13 (26.5)	36 (73.5)	
Yes	6 (14.0)	1 (14.3)	1.00	6 (14.6)	1 (16.7)	1.00	4 (11.4)	3 (21.4)	0.39	1 (7.7)	6 (16.7)	0.66
No	37 (86.1)	6 (85.7)		35 (85.4)	5 (83.3)		31 (88.6)	11 (78.6)		12 (92.3)	30 (83.3)	
**CAR T as prior BCMA-TT**	43 (86.0)	7 (14.0)		41 (87.2)	6 (12.8)		35 (71.4)	14 (28.6)		13 (26.5)	36 (73.5)	
Yes	4 (9.3)	1 (14.3)	0.55	5 (12.2)	0 (0.0)	1.00	2 (5.7)	3 (21.4)	0.13	0 (0.0)	5 (13.9)	0.31
No	39 (90.7)	6 (85.7)		36 (87.8)	6 (100.0)		33 (94.3)	11 (78.6)		13 (100.0)	31 (86.1)	
**≥ SD to prior BCMA-TT**	42 (87.5)	6 (12.5)		39 (86.7)	6 (13.3)		34 (72.3)	13 (27.7)		13 (27.7)	34 (72.3)	
Yes	20 (47.6)	4 (66.7)	0.67	20 (51.3)	2 (33.3)	0.67	16 (47.1)	8 (61.5)	0.52	9 (69.2)	15 (44.1)	0.19
No	22 (52.4)	2 (33.3)		19 (48.7)	4 (66.7)		18 (52.9)	5 (38.5)		4 (30.8)	19 (55.9)	
**≥ PR to prior BCMA-TT**	42 (87.5)	6 (12.5)		39 (86.7)	6 (13.3)		34 (72.3)	13 (27.7)		13 (27.7)	34 (72.3)	
Yes	8 (19.1)	2 (33.3)	0.59	8 (20.5)	1 (16.7)	1.00	5 (14.7)	5 (38.5)	0.11	3 (23.1)	7 (20.6)	1.00
No	34 (80.9)	4 (66.7)		31 (79.5)	5 (83.3)		29 (85.3)	8 (61.5)		10 (76.9)	27 (79.4)	
**Prior BCMA-TT < 3 months from ide-cel**	43 (87.8)	7 (14.0)		41 (87.2)	6 (12.8)		35 (71.4)	14 (28.6)		13 (26.5)	36 (73.5)	
Yes	8 (18.6)	1 (14.3)	1.00	7 (17.1)	0 (0.0)	0.57	7 (20.0)	2 (14.3)	1.00	3 (23.1)	6 (16.7)	0.68
No	35 (81.4)	6 (85.7)		34 (82.9)	6 (100.0)		28 (80.0)	12 (85.7)		10 (76.9)	30 (83.3)	

**Prior BCMA-TT < 6 months from ide-cel**	43 (87.8)	7 (14.0)		41 (87.2)	6 (12.8)		35 (71.4)	14 (28.6)		13 (26.5)	36 (73.5)	
Yes	19 (44.2)	1 (14.3)	0.22	17 (41.5)	1 (16.7)	0.38	16 (45.7)	4 (28.6)	0.34	8 (61.5)	12 (33.3)	0.10
No	24 (55.8)	6 (85.7)		24 (58.5)	5 (83.3)		19 (54.3)	10 (71.4)		5 (38.5)	24 (66.7)	
**Bridging therapy**	43 (86.0)	7 (14.0)		41 (87.2)	6 (12.8)		35 (71.4)	14 (28.6)		13 (26.5)	36 (73.5)	
Yes	37 (86.1)	6 (85.7)	1.00	34 (82.9)	6 (100.0)	0.57	31 (88.6)	11 (78.6)	0.39	12 (92.3)	30 (83.3)	0.66
No	6 (13.9)	6 (14.3)		7 (17.1)	0 (0.0)		4 (11.4)	3 (21.4)		1 (7.7)	6 (16.7)	
**≥ SD as response to bridging therapy**	28 (82.4)	6 (17.6)		25 (80.6)	6 (19.4)		28 (84.8)	8 (15.2)		12 (36.4)	21 (63.6)	
Yes	19 (67.9)	4 (66.7)	1.00	18 (72.0)	3 (50.0)	0.36	18 (72.0)	5 (62.5)	0.67	7 (58.3)	16 (76.2)	0.43
No	9 (32.1)	2 (33.3)		7 (28.0)	3 (50.0)		7 (28.0)	3 (37.5)		5 (41.7)	5 (21.8)	
**Prior autologous SCT**	43 (86.0)	7 (14.0)		41 (87.2)	6 (12.8)		35 (71.4)	14 (28.6)		13 (26.5)	36 (73.5)	
Yes	37 (86.1)	7 (100.0)	0.58	27 (90.2)	5 (83.3)	0.51	30 (85.7)	13 (92.9)	0.66	9 (69.2)	34 (94.4)	***0.036***
No	6 (13.9)	0 (0.0)		4 (9.8)	1 (16.7)		5 (14.3)	1 (7.1)		4 (30.8)	2 (5.6)	
**Prior allogeneic SCT**	43 (86.0)	7 (14.0)		41 (87.2)	6 (12.8)		35 (71.4)	14 (28.6)		13 (26.5)	36 (73.5)	
Yes	2 (4.7)	0 (0.0)	1.00	2 (4.9)	0 (0.0)	1.00	1 (2.9)	1 (7.1)	0.49	0 (0.0)	2 (5.6)	1.00
No	41 (95.3)	7 (100.0)		39 (95.1)	6 (100.0)		34 (97.1)	13 (92.9)		13 (100.0)	34 (94.4)	
**Triple-class refractory**	43 (86.0)	7 (14.0)		41 (87.2)	6 (12.8)		35 (71.4)	14 (28.6)		13 (26.5)	36 (73.5)	
Yes	39 (90.7)	6 (85.7)	0.55	37 (90.2)	5 (83.3)	0.51	31 (88.6)	13 (92.9)	1.00	13 (100.0)	31 (86.1)	0.31
No	4 (9.3)	1 (14.3)		4 (9.8)	1 (16.7)		4 (11.4)	1 (7.1)		0 (0.0)	5 (13.9)	
**Penta-refractory**	43 (86.0)	7 (14.0)		41 (87.2)	6 (12.8)		35 (71.4)	14 (28.6)		13 (26.5)	36 (73.5)	
Yes	25 (58.1)	6 (85.7)	0.23	25 (61.0)	4 (66.7)	1.00	21 (60.0)	9 (64.3)	1.00	11 (84.6)	19 (52.8)	***0.05***
No	18 (41.9)	1 (14.3)		16 (39.0)	2 (33.3)		14 (40.0)	5 (35.7)		2 (15.4)	17 (47.2)	
**Bone marrow plasma cells ≥ 50% prior to LD**	39 (90.6)	4 (9.4)		37 (90.2)	4 (9.8)		32 (74.4)	11 (25.6)		11 (25.6)	32 (74.4)	
Yes	12 (30.8)	1 (25.0)	1.00	11 (29.7)	1 (25.0)	1.00	11 (34.4)	2 (18.2)	0.46	4 (36.4)	9 (28.1)	0.71
No	27 (69.2)	3 (75.0)		26 (70.3)	3 (75.0)		21 (65.6)	9 (81.8)		7 (63.6)	23 (71.9)	

**Ide-cel cell dose (10e6)**	43 (86.0)	7 (14.0)		41 (87.2)	6 (12.8)		35 (71.4)	14 (28.6)		13 (26.5)	36 (73.5)	
< 400	21 (48.9)	2 (28.6)	0.43	21 (51.2)	1 (16.7)	0.19	15 (42.9)	7 (50.0)	0.76	6 (46.2)	16 (44.4)	1.00
≥ 400	22 (51.2)	5 (71.4)		20 (48.8)	5 (83.3)		20 (57.1)	7 (50.0)		7 (53.8)	20 (55.6)	
**Baseline ferritin at LD**	43 (86.0)	7 (14.0)		41 (87.2)	6 (12.8)		35 (71.4)	14 (28.6)		13 (26.5)	36 (73.5)	
< 400 mg/L	23 (53.5)	2 (28.6)	0.42	22 (53.7)	1 (16.7)	0.19	17 (48.6)	8 (57.1)	0.75	6 (46.2)	19 (52.8)	0.75
≥ 400 mg/L	20 (46.5)	5 (71.4)		19 (46.3)	5 (83.3)		18 (51.4)	6 (42.9)		7 (53.8)	17 (47.2)	
**Baseline CRP at LD**	43 (86.0)	7 (14.0)		41 (87.2)	6 (12.8)		35 (71.4)	14 (28.6)		13 (26.5)	36 (73.5)	
< 10.0 mg/L	39 (90.7)	5 (71.4)	0.19	39 (95.1)	4 (66.7)	*0.074*	31 (88.6)	13 (92.9)	1.00	11 (84.6)	33 (91.7)	0.6
≥ 10.0 mg/L	4 (9.3)	2 (28.6)		2 (4.9)	2 (33.3)		4 (11.4)	1 (7.1)		2 (15.4)	3 (8.3)	
**Baseline beta-2 microglobulin at LD**	34 (87.2)	5 (12.8)		33 (91.7)	3 (8.3)		28 (73.7)	10 (26.3)		10 (26.3)	28 (73.7)	
< 5.5 mg/L	29 (85.3)	4 (80.0)	1.00	29 (87.9)	1 (33.3)	*0.066*	24 (85.7)	9 (90.0)	1.00	9 (90.0)	24 (85.7)	1.00
≥ 5.5 mg/L	5 (14.7)	1 (20.0)		4 (12.1)	2 (66.7)		4 (14.3)	1 (10.0)		1 (10.0)	4 (14.3)	
**Baseline LDH at LD**	43 (86.0)	7 (14.0)		41 (87.2)	6 (12.8)		35 (71.4)	14 (28.6)		13 (26.5)	36 (73.5)	
< 225 U/L	22 (51.2)	2 (28.6)	0.42	22 (53.7)	2 (33.3)	0.42	14 (40.0)	10 (71.4)	*0.062*	4 (30.8)	20 (55.6)	0.20
≥ 225 U/L	21 (48.8)	5 (71.4)		19 (46.3)	4 (66.7)		21 (60.0)	4 (28.6)		9 (69.2)	16 (44.4)	
**Baseline ALC at LD**	43 (86.0)	7 (14.0)		41 (87.2)	6 (12.8)		35 (71.4)	14 (28.6)		13 (26.5)	36 (73.5)	
< 1.0 K/uL	21 (48.8)	4 (57.1)	1.00	19 (46.3)	4 (66.7)	0.42	18 (51.4)	7 (50.0)	1.00	6 (46.2)	19 (52.8)	0.75
≥ 1.0 K/uL	22 (51.1)	3 (42.9)		22 (53.7)	2 (33.3)		17 (48.6)	7 (50.0)		7 (53.8)	17 (47.2)	
**CrCl < 45 mL/min**	43 (86.0)	7 (14.0)		41 (87.2)	6 (12.8)		35 (71.4)	14 (28.6)		13 (26.5)	36 (73.5)	
Yes	6 (13.9)	0 (0.0)	0.58	4 (9.8)	2 (33.3)	0.16	3 (8.6)	3 (21.4)	0.33	1 (7.7)	5 (13.9)	1.00
No	37 (86.1)	7 (100.0)		37 (90.2)	4 (66.7)		32 (91.4)	11 (78.8)		12 (92.3)	31 (86.1)	
**LVEF < 45%**	43 (86.0)	7 (14.0)		41 (87.2)	6 (12.8)		35 (71.4)	14 (28.6)		13 (26.5)	36 (73.5)	
Yes	3 (7.0)	0 (0.0)	1.00	3 (7.3)	0 (0.0)	1.00	1 (2.9)	2 (14.3)	0.19	0 (0.0)	3 (8.3)	0.56
No	40 (93.0)	7 (100.0)		38 (92.7)	6 (100.0)		34 (97.1)	12 (85.7)		13 (100.0)	33 (91.7)	
**KarMMa ineligible by comorbidities***	42 (85.7)	7 (14.3)		41 (89.1)	5 (10.9)		35 (71.4)	14 (28.6)		13 (37.1)	35 (72.9)	

Yes	5 (11.9)	1 (14.3)	1.00	6 (14.6)	0 (0.0)	1.00	4 (11.8)	2 (14.3)	1.00	3 (23.1)	3 (8.6)	0.32
No	37 (88.1)	6 (85.7)		35 (85.4)	5 (100.0)		30 (88.2)	12 (85.7)		10 (76.9)	32 (91.4)	

CRS, cytokine release syndrome; ICANS, immune-effector cell-associated neurotoxicity syndrome; CR, complete response; ORR, overall response rate; G, grade; PR, partial response; *P,* p-value by chi-square test; EM, extramedullary; ECOG PS, Eastern Cooperative Oncology Group Performance Status; R-ISS, Revised International Staging System; FISH, fluorescence in-situ hybridization; ADC, antibody-drug conjugate; BCMA-TT, BCMA-targeted therapy; CAR T, chimeric antigen receptor T-cell; SD, stable disease; SCT, stem cell transplantation; LD, lymphodepletion; CRP, C-reactive protein; LDH, lactate dehydrogenase; ALC, absolute lymphocyte count; CrCl, creatinine clearance; LVEF, left ventricular ejection fraction

*All patients would have been ineligible for KarMMa trial due to prior BCMA-TT. This indicates those ineligible for additional reasons.
